# Hypokalemic paralysis associated with cystic disease of the kidney: case report

**DOI:** 10.1186/1471-2369-12-16

**Published:** 2011-04-18

**Authors:** Champika SSSK Gamakaranage, Chaturaka Rodrigo, Saroj Jayasinghe, Senaka Rajapakse

**Affiliations:** 1 University Medical Unit, National Hospital, Colombo 08, Sri Lanka; 2 Department of Clinical Medicine, Faculty of Medicine, University of Colombo, Colombo 08, Sri Lanka

**Keywords:** hypokalemia, renal tubular acidosis, medullary cystic kidney, renal cysts

## Abstract

**Background:**

Severe hypokalemia is known to cause muscle paralysis, and renal tubular acidosis is a recognized cause. Cystic disease of the kidney is associated with severe hypokalemia.

**Case presentation:**

We report a 33-year-old male patient who presented with generalized limb weakness caused by severe hypokalemia due to renal tubular acidosis, who was found to have renal medullary cysts.

**Conclusion:**

The association of cystic renal disease with hypokalemia, and the possible pathophysiological basis of the development of renal cysts in patients with severe hypokalemia, are discussed.

## Background

Severe hypokalemia is known to result in muscle paralysis[1], and renal tubular acidosis (RTA) is a recognized cause[2,3]. RTA is a condition where acid excretion by the kidneys is impaired, resulting in systemic acidosis. The classical type of RTA is the 'distal' or Type 1. Renal potassium loss occurs in Type 1 RTA, which results in hypokalemia[4]. There are numerous causes and associations of Type 1 RTA, and the condition can be hereditary, congenital, acquired or idiopathic[4]. RTA and severe hypokalemia have been associated with medullary sponge kidney[5] and other cystic conditions of the kidney. Nephrocalcinosis is also often associated[4]. The reason for formation of renal cysts, either in the classical form of medullary sponge kidney, or other variants, is unclear. We report a patient who presented with severe hypokalemia due to renal tubular acidosis who was found to have cystic renal disease, and discuss the possible pathogenesis of cyst formation in the kidney.

## Case presentation

A 33-year-old male farmer presented with a history of excessive thirst, weight loss, and increased urine output over the preceding two months. He then developed generalized body weakness, associated with vomiting which occurred 3-5 times a day, over the past 2 weeks prior to admission. The weakness, which was predominantly in the limbs and the neck, worsened over the course of the next two weeks, and on admission he was bed bound. At the time of presentation his motor power was grade 2 in all limbs (unable to move against gravity, movement possible with gravity eliminated[6]), and he was unable to move his neck. The limbs were hypotonic with absent reflexes, and muscle tenderness was present. However, there was no respiratory difficulty, diplopia or dysarthria. He retained his bladder and bowel control. His height was 156 cm and weight was 52 kg (BMI of 21.4 kg/m^2^). Cardiovascular, respiratory and abdominal examination did not reveal any abnormalities. While this acute episode was dramatic in onset, it was also noted that over the last 2 years he had intermittently complained of ill health, pain in the legs, backache and body pains, and had been off work frequently.

He had no history of any other significant medical or psychiatric comorbidities. There was no other significant illness that required hospitalization or long term treatment during his neonatal period or childhood. He had not been on any long term medication or indigenous medicine. There was no significant family history. He had not used any addictive substances.

His serum potassium level was less than 1 mEq/l (3.5-5.1) at that time of peak paralysis; serum sodium was 135 mEq/l (135-148), chloride 96 mEq/l (95-105). The electrocardiogram at this stage showed flattened P waves, and U waves. His serum creatinine readings were between 80 - 120 μmol/l (60-120) and the random blood sugar was 102 mg/dl (<200). The estimated glomerular filtration rate (eGFR) was between 64-90 ml/min/m^2 ^based on these values. He had neutrophil leucocytosis (total count 18,800 cells/μl with 90% neutrophils). The hemoglobin and platelet counts were within the reference range.

The priority at this stage was correction of his serum potassium. With intravenous replacement of potassium, the muscle power and the overall clinical condition of the patient improved rapidly.

We investigated him for a cause of hypokalemia. The main reasons for hypokalemia are excessive losses (renal or gastrointestinal loss) or redistribution within body compartments. This patient had vomiting and polyuria, hence loss through the gastrointestinal tract or kidneys was considered. Given the protracted history for 2 years, associated with polyuria, polydipsia and weight loss, we considered renal potassium loss to be more likely. Spot urine analysis showed urinary potassium of 24 mEq/l(13-62) and sodium of 120 mEq/l. The patient had a 24 hour urine volume of 3500 ml, and despite severe hypokalemia he was losing potassium in his urine. A subsequent 24-hour urinary potassium level confirmed an abnormally excessive urinary potassium loss of 80 mmol/24 hours despite severe hypokalemia (reference range: 40-100).

Urinalysis revealed a urine pH of 6.5 and 7.5 on two occasions. There were a few calcium oxalate crystals and 2-4 red cells per high power field. There was also a mild hypercalciuria, with a 24-hour urinary calcium excretion of 0.134 mmol/kg (upper limit 0.1 mmol/kg/day). The rest his urinalysis was normal and there was no glycosuria or proteinuria.

At the time of peak paralysis, the arterial blood gas showed a pH of 7.45 (7.35-7.45), pCO_2 _of 22.4 mmHg (36-44), pO_2 _of 129 mmHg (80-100), Oxygen saturation of 99%, Bicarbonate of 15.6 mmol/l (22-28), and a base excess of (-)8.6 mEq/l. This picture was suggestive of a compensated metabolic acidosis. He was admitted to the intensive care unit and daily arterial blood gases were performed. These continued to show metabolic acidosis (pH: 7.20-7.25). Plasma anion gap was 12.2 mEq/l (10-14). Thus this patient had normotensive hypokalemia with a normal anion gap (hyperchloremic) metabolic acidosis, and persistently alkaline urine (his urine pH was >5.5 despite metabolic acidosis). The urine anion gap was positive (U_Na _120 + U_K _24 - U_Cl _116 = 28). These findings are compatible with renal tubular acidosis (RTA), most likely distal/type 1 RTA (dRTA 1).

While further investigating for a cause for the RTA, renal ultrasound showed bilaterally enlarged kidneys with multiple cysts of varying sizes. Cortical echogenicity was increased with altered cortico-medullary demarcation.

**Figure 1:**
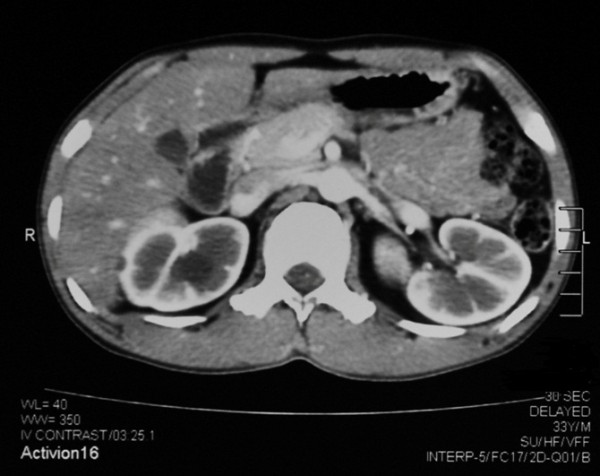
**Contrast-enhanced CT scan abdomen showing multiple medullary cysts in both kidneys**.

This was investigated further with a computed tomography-intravenous urogram (CT IVU) which revealed enlarged (right -125 mm, left -123 mm) kidneys with variable sized multiple thin walled medullary cysts with no septae in both kidneys (figure 1). The largest cyst measured 30 mm × 25 mm. Fine calcified foci of 1-2 mm were seen in cortices of both kidneys. Similar cysts were not observed in the liver, pancreas or in any other solid organ.

The creatine kinase level in this patient was high (initially 704 IU/l and increased to 1262 IU/L subsequently, reference range: 60-300) probably due to rhabdomyolysis associated with hypokalemia and RTA [5,7], although this did not result in acute kidney injury (AKI).

Liver transaminases were elevated [AST -151 U/l (10-40) and ALT - 208 U/l (10-40), which could be due to many reasons, related or unrelated to renal tubular acidosis. The possibility of Wilsons disease was considered as it may also present as renal tubular acidosis[7]. However, ophthalmological assessment excluded Kaiser-Fleisher (KF) rings in this patient. Chronic active hepatitis is also known to be associated with renal tubular acidosis and hypokalemia[8].

## Conclusions

The most common causes of hypokalemic paralysis include thyrotoxic periodic paralysis, renal tubular acidosis, and hypokalemic periodic paralysis[1]. Cystic disease of the kidney has long been associated with hypokalemia and sometimes with renal tubular acidosis. One of the classical associations between cystic disease of the kidney, renal tubular acidosis and hypokalemia is medullary sponge kidney (MSK)[5]. MSK is a poorly understood disease[9]. Patients are usually asymptomatic; those with symptoms usually present with renal or ureteric colic, haematuria and polyuria. Often, a patient with a medullary sponge kidney may undergo decades of suffering with infections and pain before the diagnosis is made. When a diagnosis is made, it is more likely to be an incidental finding. The changes seen in MSK are thought to reflect a developmental or congenital abnormality, although the true underlying defect is unknown[10]. There is no firm evidence of genetic transmission, yet there are some families that appear to show an autosomal dominant inheritance[5,11]. Medullary sponge kidney is known to be associated with hypokalemia, nephrocalcinosis and RTA[5,12,13].

The diagnosis of MSK is typically made by intravenous urogram[10,14]. It is characterized by ectasia of the papillary collecting ducts in the medulla. These converge at the tips of pyramids where all or many of the pyramids are involved. On intravenous urogram these cystic dilatations lead to the appearance of a 'brush' radiating outward from some or all of the calyces ('bouquet of flowers' appearance) due to collection of contrast in the dilated ducts[15]. Enlargement of the pyramids and intra-ductal concretions are also often seen. Calcium stones, if present, are typically small and, almost pathognomonically, occur in clusters limited to the affected calyces. MSK is essentially a radiological diagnosis, with numerous reported associations. The long term prognosis is excellent, although obstructive uropathy due to calculi can result in transient reductions in glomerular filtration rate. Rarely calculi may cause recurrent obstruction and infection in which can progress to chronic kidney disease.

In this patient, renal tubular acidosis resulting in severe hypokalemia appeared to be the primary diagnosis to explain his clinical symptoms. Although renal cysts were identified on radiological imaging, the classical features of MSK were not found (see above). Hence, other causes of cystic disease were also considered. The cysts were medullary in location, and thus not in keeping with polycystic kidney disease.

Torres et al[16] had suggested that, at least in a proportion of patients, renal cyst formation may be a *result *of severe hypokalemia rather than a simple association. The study compared patients with aldosteronism and hypokalemia with controls with essential hypertension. Renal cyst formation was significantly more common in patients with hypokalemia and aldosteronism, and the extent of cyst development correlated with lower potassium levels. Furthermore, removal of the adrenal adenomas resulted in regression of the cysts which also corresponded to the normalization of serum potassium levels.

Cyst formation is thought to occur due to enhanced growth and proliferation of the epithelial cells lining the cysts[17]. Under experimental conditions, hypokalemia stimulates protein synthesis and cell division[18,19]. This evidence supports the postulate that severe hypokalemia promotes the formation of renal cysts. We suggest that the renal cysts detected in this patient developed as a result of profound hypokalemia, and is neither a simple association, nor the primary abnormality resulting in RTA and hypokalemia. We also propose that hypokalemia induced tissue proliferation may, at least in some cases, be the etiological factor for the often described association of renal tubular acidosis and cystic disease of the kidney. If this were the case, it is probable possible that long term correction of hypokalemia would result in regression of the cysts. We intend to follow this patient up to determine this.

## Consent

Written informed consent was obtained from the patient for this case to be published.

## Abbreviations

MSK: Medullary Sponge Kidney; RTA: Renal Tubular Acidosis; CT-IVU: Computerised tomography-intravenous urogram; BMI: body mass index

## Competing interests

The authors declare that they have no competing interests.

## Authors' contributions

CSSKG, CR and SR researched the background literature on the case and wrote the first draft. SJ contributed towards the discussions and analysis of the case. SR wrote the final draft. All authors reviewed the final manuscript.

## Authors' information

CSSKG (MBBS) is registrar in general medicine in the University Medical Unit, National Hospital, Colombo, Sri Lanka. CR(MBBS) is Lecturer in Medicine, and SJ(MD, FRCP, MD) and SR(MD, FRCP Edin, FACP) are Professors in Medicine, in the Department of Clinical Medicine, Faculty of Medicine, University of Colombo, Sri Lanka.

## Pre-publication history

The pre-publication history for this paper can be accessed here:

http://www.biomedcentral.com/1471-2369/12/16/prepub
